# Copper-catalyzed enantioselective conjugate addition of organometallic reagents to challenging Michael acceptors

**DOI:** 10.3762/bjoc.16.24

**Published:** 2020-02-17

**Authors:** Delphine Pichon, Jennifer Morvan, Christophe Crévisy, Marc Mauduit

**Affiliations:** 1 Université de Rennes, Ecole Nationale Supérieure de Chimie de Rennes, CNRS, ISCR – UMR 6226, F-35000 Rennes, France

**Keywords:** acylimidazole, *N*-acyloxazolidinone, *N*-acylpyrrole, *N*-acylpyrrolidinone, aldehyde, amide, copper catalysis, electron-deficient alkenes, enantioselective conjugate addition, Michael acceptor, thioester

## Abstract

The copper-catalyzed enantioselective conjugate addition (ECA) of organometallic nucleophiles to electron-deficient alkenes (Michael acceptors) represents an efficient and attractive methodology for providing a wide range of relevant chiral molecules. In order to increase the attractiveness of this useful catalytic transformation, some Michael acceptors bearing challenging electron-deficient functions (i.e., aldehydes, thioesters, acylimidazoles, *N*-acyloxazolidinones, *N*-acylpyrrolidinones, amides, *N*-acylpyrroles) were recently investigated. Remarkably, only a few chiral copper-based catalytic systems have successfully achieved the conjugate addition of different organometallic reagents to these challenging Michael acceptors, with excellent regio- and enantioselectivity. Furthermore, thanks to their easy derivatization, the resulting chiral conjugated products could be converted into various natural products. The aim of this tutorial review is to summarize recent advances accomplished in this stimulating field.

## Introduction

Generating high molecular complexity and controlling multiple stereogenic centers in a minimum number of steps is nowadays one of the most important challenges in organic chemistry for the synthesis of complex chiral molecules. The transition metal (TM)-catalyzed enantioselective conjugate addition (ECA) of nucleophiles to electron-deficient alkenes (Michael acceptors) is one of the most relevant and versatile methods to achieve this goal [1–4]. Among the plethora of metals studied, copper-based catalytic systems proved to be highly efficient for the conjugate addition of various organometallic reagents, such as diorganozinc, triorganoaluminium, and Grignard reagents to Michael acceptors. In that respect, since the pioneering example reported by Alexakis and co-workers in 1993 [5], a wide range of cyclic and acyclic electron-deficient alkenes, such as α,β-unsaturated ketones, esters, nitriles, sulfones, or nitroolefines, was intensively studied, leading to the expected 1,4-products in excellent yields and remarkable enantioselectivities. More recently, tremendous breakthroughs were achieved in this field, notably by the formation of all-carbon quaternary chiral centers [6] and the challenging 1,6-, 1,8-, or 1,10-selective addition to cyclic or aliphatic polyenic substrates [7–9]. Furthermore, Cu ECA transformations were also successfully applied to the synthesis of natural products [10]. Nevertheless, it is worth to underline that the choice of the electron-withdrawing group (EWG) on the Michael substrates appears not so simple. First, the hardness of the involved organometallic reagents has to be considered in order to overcome or limit undesirable side reactions. Although the main role of copper is to form a transient organocuprate reactive species with the hard nucleophiles to avoid the formation of the nondesired 1,2-product, some Grignard or aluminium reagents remain too reactive and incompatible with some electron-withdrawing functions. In contrast, some organometallic reagents, such as dimethylzinc, are poorly reactive and require a higher electrophilicity of the Michael acceptors to provide the desired conjugated products. Second, in order to be attractive for the total synthesis of relevant molecules, the involved EWG should preferably allow readily applicable and practicable postfunctionalizations [10].

Among the plethora of studied Michael acceptors, α,β-unsaturated aldehydes, thioesters, acylimidazoles, *N*-acyloxazolidinones, *N*-acylpyrrolidinones, amides, and *N*-acylpyrroles have been scarcely investigated in Cu ECA despite their usefulness for postfunctionalizations. This tutorial review aims to describe the early examples and recent advances in copper-catalyzed asymmetric conjugate additions of dialkylzinc, Grignard, or trialkylaluminium reagents toward those challenging substrates and their fruitful application in the total synthesis of natural products.

## Review

### Enantioselective conjugate addition to challenging Michael acceptors

#### Copper-catalyzed ECA to α,β-unsaturated aldehydes

**Scheme 1:**
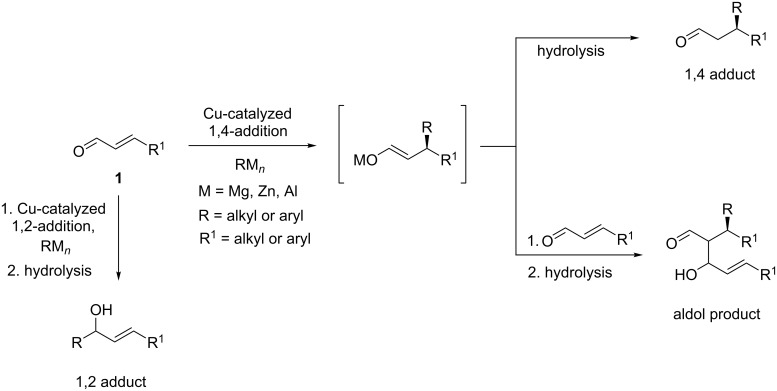
Competitive side reactions in the Cu ECA of organometallic reagents to α,β-unsaturated aldehydes.

Nowadays, β-substituted enals represent probably the most challenging Michael acceptors in the copper-catalyzed ECA of organometallic reagents [11–13]. This challenge is reinforced by the fact that the resulting chiral β-functionalized aldehydes are considered as an important motif that is ubiquitous in numerous natural molecules. However, as depicted in Scheme 1, due to their stronger reactivity than that of usual esters or ketones, a competitive 1,2-addition to the carbonyl function of enals could occur, leading to the corresponding alcohol as a byproduct. Moreover, even if the 1,4-addition is favored, thanks to the copper/ligand catalytic species, the resulting metallic enolate intermediate can also react with the starting material to form the aldol byproduct, significantly altering the yield of the expected 1,4-product (Scheme 1).

**Scheme 2:**
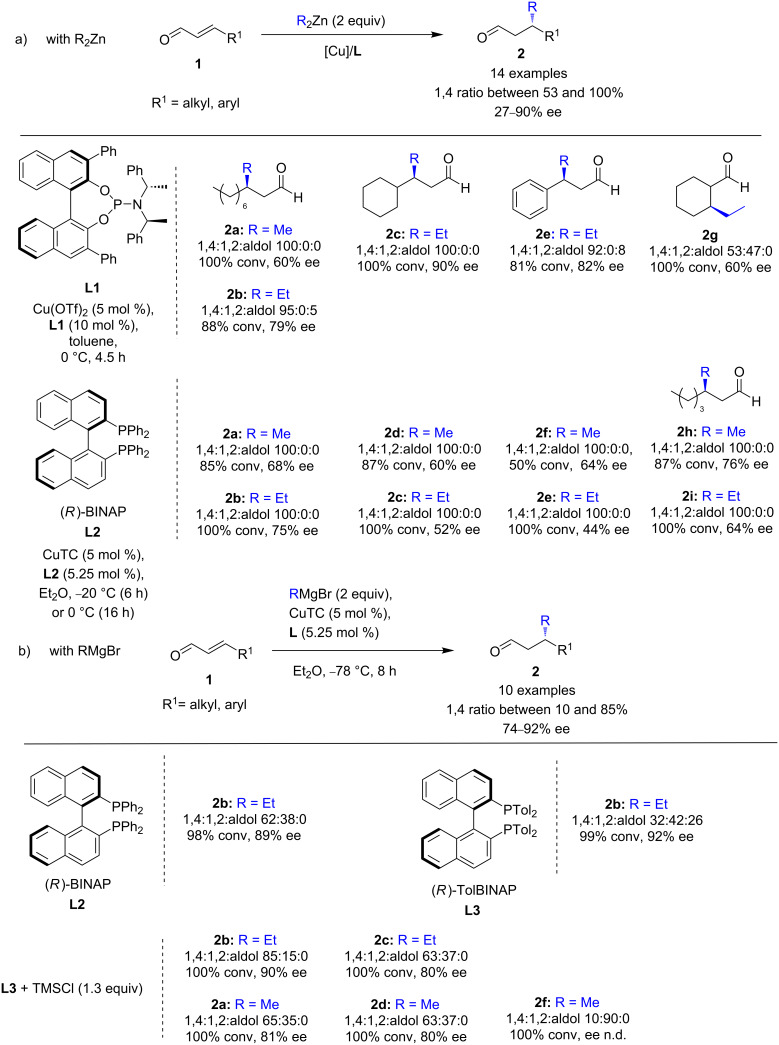
Cu-catalyzed ECA of α,β-unsaturated aldehydes with phosphoramidite- (a) and phosphine-based ligands (b).

The first successful copper-catalyzed ECA to α,β-unsaturated aldehydes with organozinc and Grignard reagents was reported by Alexakis and co-workers in 2010 [14]. After screening various chiral phosphine-based ligands, the combinations of either phosphoramidite **L1** with Cu(OTf)_2_, or (*R*)-BINAP (**L2**) with copper thiophenecarboxylate (CuTC) appeared to be the most efficient for the addition of Et_2_Zn to a variety of cyclic and acyclic aldehydes **1**. High 1,4-regioselectivities and promising stereoselectivities ranging from 27 to 90% ee were achieved (Scheme 2a). It is noteworthy that the addition of dimethylzinc was also successfully achieved, as the desired 1,4-methylated products were exclusively formed in moderate to good yields, with ee values of up to 76%. When the conjugate addition was performed with Grignard reagents, significant amounts of 1,2-products and enols were formed, despite the use of cryogenic conditions. (*R*)-BINAP (**L2**) gave the best regio- and enantioselectivity, with 62% of the 1,4-product and 89% ee with EtMgBr (Scheme 2b). To overcome the low regioselectivity, the authors took into account previous works showing that the 1,4-regioselectivity in the addition of cuprates to enals could be improved in the presence of a slight excess of TMSCl [15–19]. Indeed, using TMSCl in combination with (*R*)-TolBINAP (**L3**), a promising 85% regioselectivity was observed, without altering the enantioselectivity (90% ee), whereas only 32% of the desired 1,4- product was obtained without TMSCl [20]. With those optimized conditions, various enals and Grignard reagents were screened. Nevertheless, despite the presence of TMSCl, the 1,4:1,2 ratio varied significantly (from 85:15 to 10:90), while the level of enantioselectivity remained relatively good, reaching up to 90%.

**Scheme 3:**
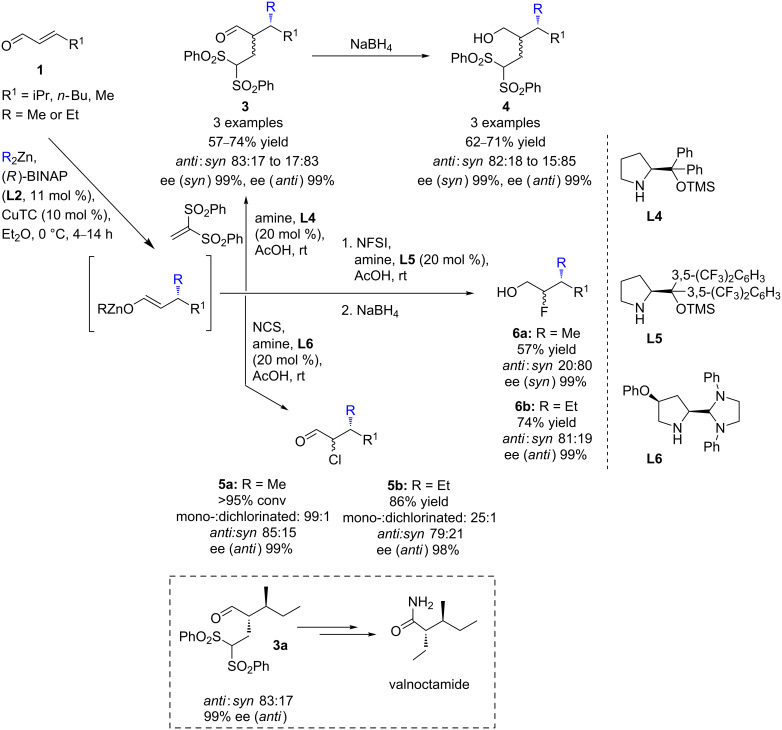
One-pot Cu-catalyzed ECA/organocatalyzed α-substitution of enals.

Following this, Alexakis and Quintard invented an efficient stepwise one-pot copper-catalyzed asymmetric ECA/organocatalyzed α-substitution of enals [21]. By using (*R*)-BINAP (**L2**)/CuTC in combination with chiral prolinol derivatives **L4**–**6** as organocatalysts, various α,β-functionalized aldehydes were synthesized in good isolated yields (57–74%) and remarkable enantioselectivity (99%) from diethylzinc or dimethylzinc as nucleophiles and vinyl sulfones as electrophiles (Scheme 3). Of note, both the *anti-* and the *syn*-product could be predominantly formed (with a *anti*:*syn* ratio from 83:17 to 15:85), and no diastereocontrol occurred in the absence of the organocatalyst. Interestingly, this simple protocol was successfully applied to the enantioselective synthesis of valnoctamide, a commercialized mild tranquilizer. Finally, this methodology was extended to the sequential Michael/halogenation reaction using NFSI or NCS as electrophiles, with similar efficiency.

**Scheme 4:**
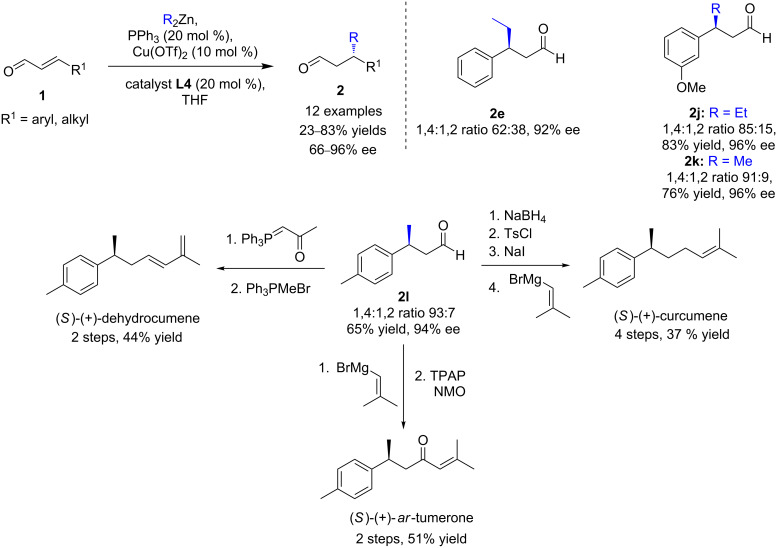
Combination of copper and amino catalysis for enantioselective β-functionalizations of enals.

Similarly, a cocatalyzed enantioselective β-functionalization of enals was developed by Córdova, Ibrahem, and co-workers in 2011 (Scheme 4) [22]*.* By mixing high catalytic loadings of Cu(OTf)_2_, PPh_3_, and TMS-protected diarylprolinol **L4**, the conjugate addition of Et_2_Zn or Me_2_Zn to various β-substituted enals proved to be highly enantioselective (ee up to 96%), but moderate to good 1,4:1,2 ratios were obtained (51:49 to 97:3). Of note, chiral phosphines were also screened, but without any improvement of selectivity. Furthermore, this methodology was then efficiently applied to the total synthesis of several bisabolane sesquiterpenes, which exhibited anticancer and antimicrobial activities or are employed as ingredients in perfumes and cosmetics (Scheme 4).

**Scheme 5:**
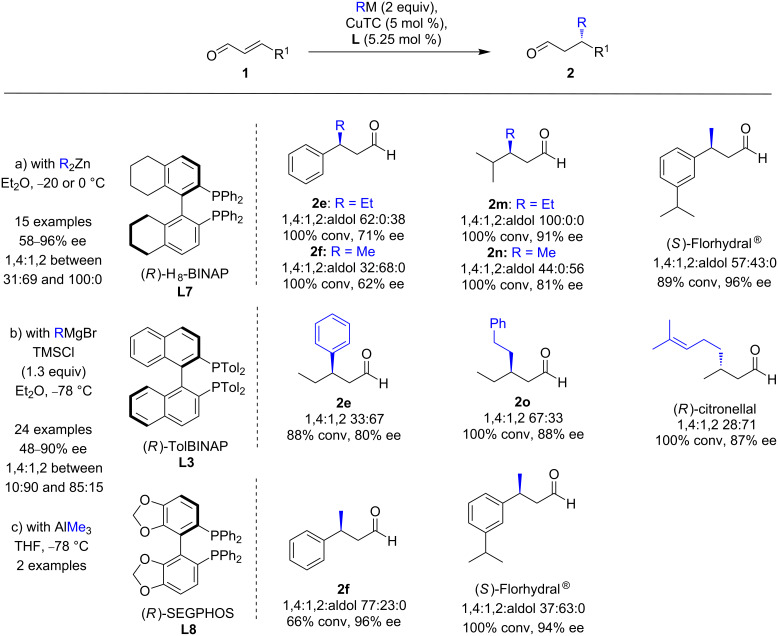
Optimized conditions for the Cu ECAs of R_2_Zn, RMgBr, and AlMe_3_ with α,β-unsaturated aldehydes.

The last report on ECAs of enals [23] was disclosed in 2016 by Alexakis and co-workers [24]. They achieved to develop three sets of optimized conditions for the CuTC-catalyzed conjugate addition of diorganozinc compounds, Grignard, and triorganoaluminium reagents to α,β-unsaturated aldehydes (Scheme 5). With diethyl- and dimethylzinc, and in the presence of the most efficient chiral ligand (*R*)-H_8_-BINAP (**L7**), moderate to excellent regioselectivities (1,4:1,2 ratios up to 100:0) were observed, and the desired 1,4-products were formed with remarkable enantioselectivities (58 to 96% ee). With Grignard reagents, the best ee values (45 to 90%) were obtained with (*R*)-TolBINAP (**L3**), but despite the presence of TMSCl, the regioselectivities remained modest, with a highest 1,4:1,2 ratio of 85:15. At last, (*R*)-SEGPHOS (**L8**) promoted the conjugate addition of Me_3_Al to cinnamaldehyde, with a remarkable 96% ee and a moderate 1,4:1,2 ratio. However, albeit no trace of aldol byproduct was detected, the reaction was incomplete (66% conversion). The use of TMSCl improved the conversion to 88%, but this was detrimental to the enantiocontrol (8% ee). These methodologies were applied to the straightforward synthesis of valuable (*R*)-citronellal and (*S*)-Florhydral^®^, which were obtained with excellent enantioselectivities (87 and 96%, respectively). However, moderate to significant amounts of the 1,2-products were also formed.

As highlighted by these pioneering works, the direct copper-catalyzed conjugate addition of organometallic reagents to α,β-unsaturated aldehydes still remains an important challenge. Albeit some promising excellent regioselectivities and high enantioselectivities were achieved, this was often limited to a few organometallic reagents/enal substrates, as mentioned above. In that respect, indirect pathways were developed as alternative strategies, involving electron-deficient functions that can subsequently easily be converted to aldehydes.

#### α,β-Unsaturated thioesters

**Scheme 6:**
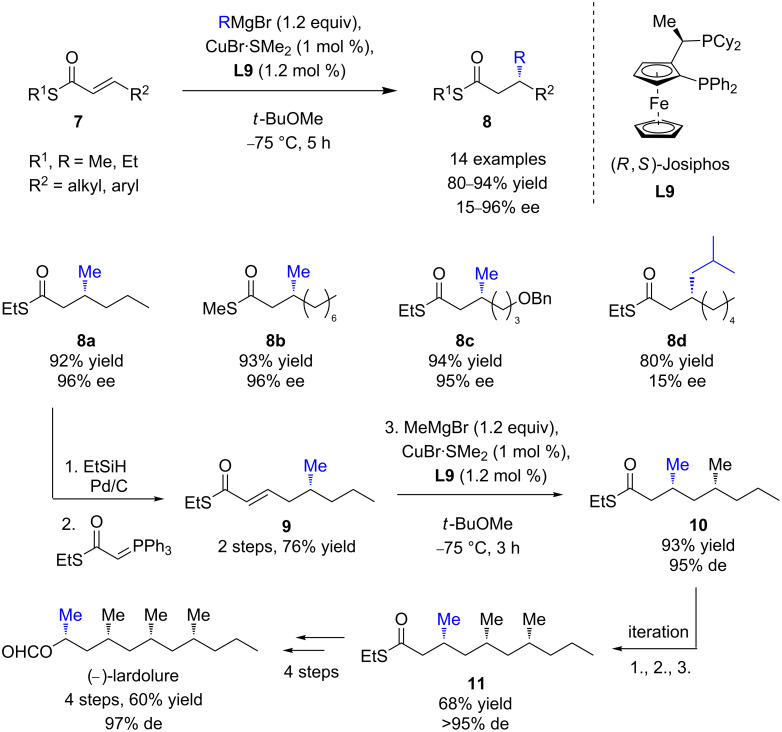
CuECA of Grignard reagents to α,β-unsaturated thioesters and their application in the asymmetric total synthesis of (–)-lardolure.

In 2005, Feringa, Minnaard, and co-workers were the first to report the ECA of Grignard reagents to α,β-unsaturated thioesters [25]. Advantageously, the latter were also readily accessible but significantly more reactive than α,β-unsaturated esters. Indeed, the thioester fragments featured a reduced electron delocalization compared to oxoesters, which resulted in a higher reactivity in conjugate additions, even with the less reactive MeMgBr. As depicted in Scheme 6, excellent yields and remarkable enantioselectivities (up to 96%) were obtained in ECAs of linear aliphatic Grignard reagents (in particular MeMgBr) to a wide range of substrates, catalyzed by CuBr∙SMe_2_/(*R*,*S*)-Josiphos (**L9**).

**Scheme 7:**
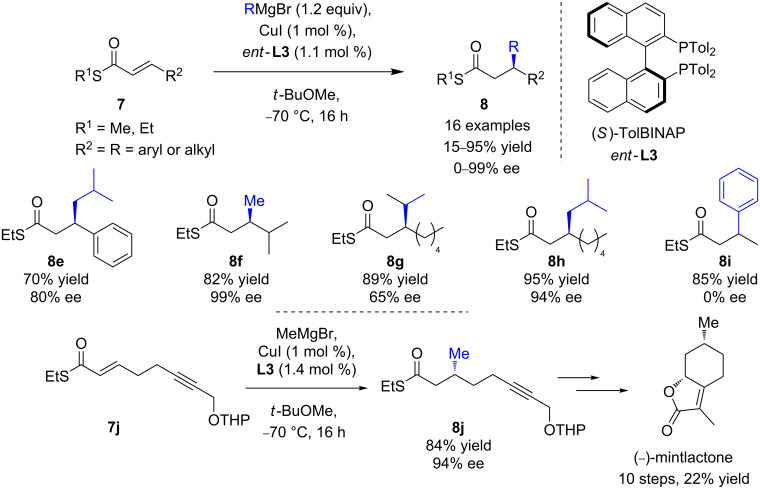
Improved Cu ECA of Grignard reagents to α,β-unsaturated thioesters, and their application in the asymmetric total synthesis of (−)-mintlactone.

However, the catalytic system was poorly selective toward sterically hindered organomagnesium nucleophiles (15–25% ee). The synthetic versatility of the thioester function was illustrated in the synthesis of (−)-lardolure (26% overall yield over 12 steps) via a relevant diastereoselective and enantioselective iterative route, affording the highly desirable deoxypropionate moiety in high 97% de. The Josiphos (**L9**)/CuBr∙SMe_2_ catalytic system was also efficient to promote the ECAs of MeMgBr to the less reactive aromatic α,β-unsaturated thioesters (ee values up to >99%) [26]. In order to extend their methodology to less reactive bulky Grignard reagents and/or substrates, a catalytic system of wider application, involving (*S*)-TolBINAP (*ent*-**L3**)/CuI was developed by the same authors [26]. The expected 1,4-products were isolated in good yields and moderate to excellent enantioselectivities (up to 99% ee, Scheme 7), depending on the steric hindrance of the reagent. Unfortunately, the addition of PhMgBr remained unsuccessful. This powerful catalytic protocol was illustrated by Bates and Sridhar in the enantioselective total synthesis of (−)-mintlactone [27]. The key step, furnishing the β-methylated thioester **8j**, was accomplished in a good yield of 84% and a high ee of 94% (Scheme 7).

**Scheme 8:**
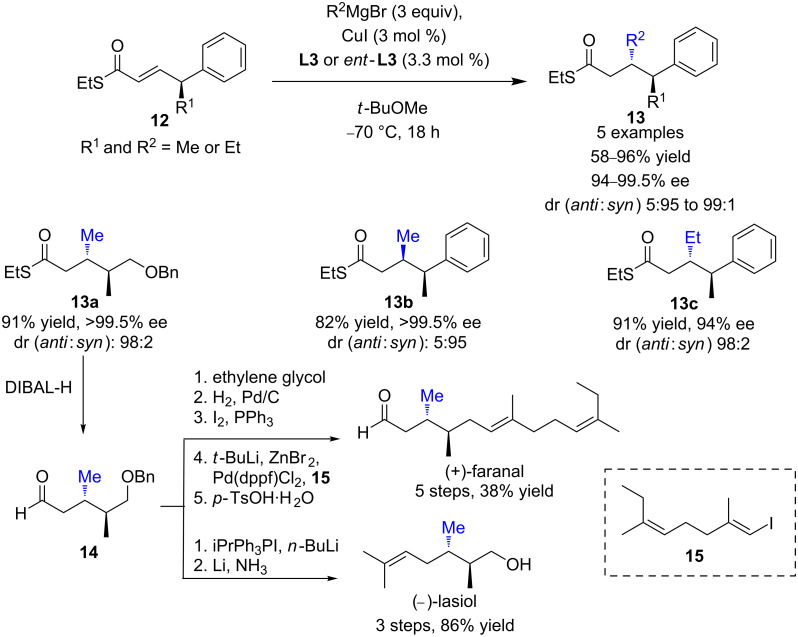
Catalytic enantioselective synthesis of vicinal dialkyl arrays via Cu ECA of Grignard reagents to γ-substituted α,β-unsaturated thioesters.

In 2008, Feringa and Minnaard evaluated the ECA of Grignard reagents to γ-substituted α,β-unsaturated thioesters that could lead to vicinal (i.e., 1,2-relation) dialkyl arrays, a highly desirable moiety that is ubiquitous in a wide range of natural products [28]. As depicted in Scheme 8, TolBINAP (**L3**)/CuI, which appeared to be a better catalytic system than Josiphos (**L9**)/CuBr∙SMe_2_, afforded either the *syn* or *anti* 1,4 product **13** in good isolated yields and excellent diastereoselectivities and enantioselectivities (dr up to 99:1 and ee up to >99.5). The value of the protocol was successfully illustrated through the enantioselective total syntheses of (−)-lasiol and (+)-faranal, two useful natural pheromones.

**Scheme 9:**
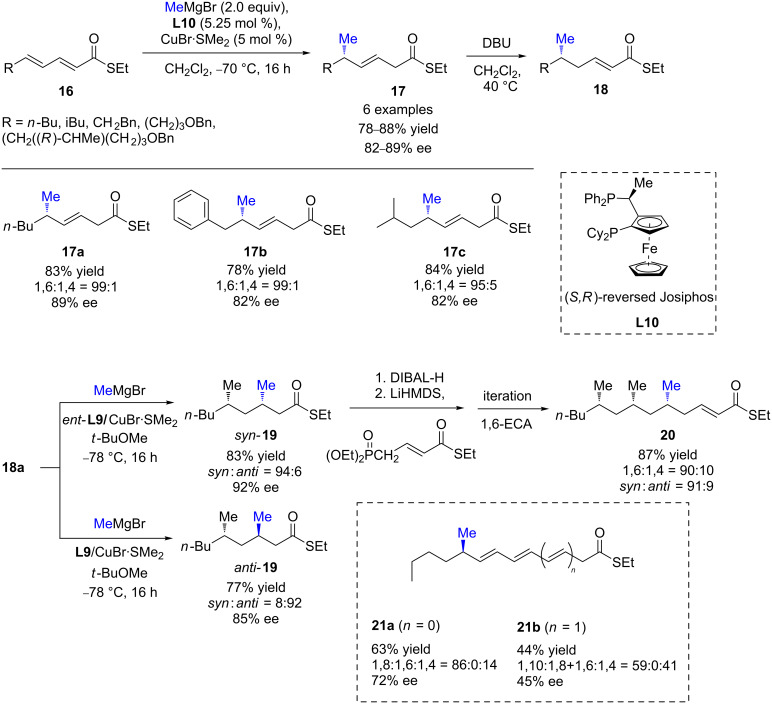
1,6-Cu ECA of MeMgBr to α,β,γ,δ-bisunsaturated thioesters: an iterative approach to deoxypropionate units.

Shortly after, Feringa, Minnaard, and co-workers demonstrated the efficiency of (*S*,*R*)-reversed Josiphos (**L10**) in the copper-catalyzed 1,6-ECA of MeMgBr to α,β,γ,δ*-*bisunsaturated thioesters [29–30]. The expected 1,6-products were selectively formed (the 1,6:1,4 ratio ranged from 85:15 to 99:1) in high yields (78–88%) and good enantioselectivities (82–89%, Scheme 9). It is worth to note that this protocol failed in the case of linear dienoates [29]. Interestingly, after a subsequent reconjugation step in the presence of DBU, the resulting enantioenriched γ-methylated α,β-unsaturated thioester **18a** was subsequently reacted in a 1,4-ECA reaction catalyzed by Josiphos (**L9**)/CuBr·SMe_2_. Using both enantiomers of the chiral ligand, either *anti*- or *syn*-1,3-deoxypropionate units were produced in good yields and excellent enantioselectivities (85–92% ee). Furthermore, an iterative procedure was also performed leading to all-*syn* or *anti*/*syn*-5,7,9-stereotriads, with high yields and stereoselectivity. This methodology was also tested on linear polyenic thioesters [9]. The challenging 1,8- and 1,10-products **21a**/**b** were obtained, but the stereoselectivity dropped when the distance between the reacting olefin and the ester function was increased (1,8-ECA 72% ee*;* 1,10-ECA 45% ee). However, the regioselectivity (59–86%) and yield (44–63%) remained decent.

**Scheme 10:**
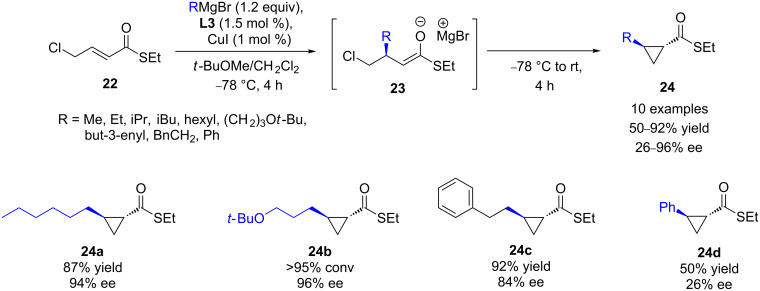
Tandem Cu ECA/intramolecular enolate trapping involving 4-chloro-α,β-unsaturated thioester **22**.

The efficiency of TolBINAP (**L3**)/CuI was also demonstrated in the ECA of Grignard reagents to the 4-chloro-α,β-unsaturated thioester **22** [31]. Interestingly, the presence of the internal chloro leaving group allowed a powerful tandem conjugate addition–enolate trapping that led to valuable *trans*-1-alkyl-2-substituted cyclopropanes (Scheme 10). Various Grignard reagents were used, affording the corresponding cyclopropanes in moderate to high yields (50–92%) and good to excellent ee values (70–96%), except for PhMgBr (26% ee).

**Scheme 11:**
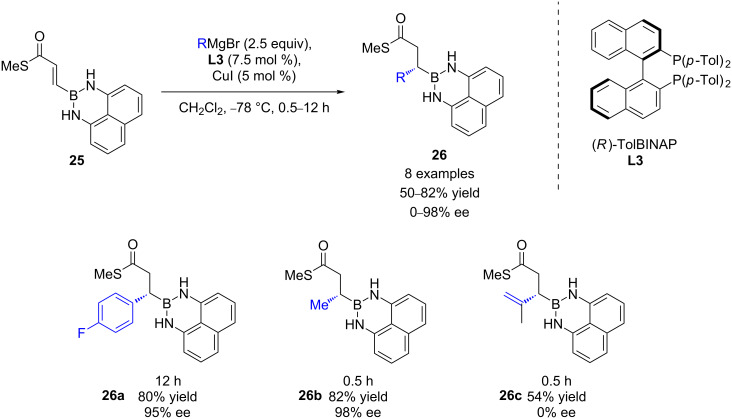
Cu ECA of Grignard reagents to 3-boronyl α,β-unsaturated thioesters.

In 2010, Hall and Lee described a successful synthesis of enantioenriched boronate derivatives through catalytic ECA of Grignard reagents to 3-boronyl α,β-unsaturated thioesters (Scheme 11) [32]. By applying an **L3**/CuI catalytic protocol previously developed by Feringa and Minnaard, MeMgBr and a range of aromatic Grignard reagents were selectively introduced, leading to the expected 1,4-products in high yields (50–82%) and ee values (82–98%). Unfortunately, *ortho*-substituted aromatic or hindered alkenyl reagents led to the corresponding products without showing any enantioselectivity.

**Scheme 12:**
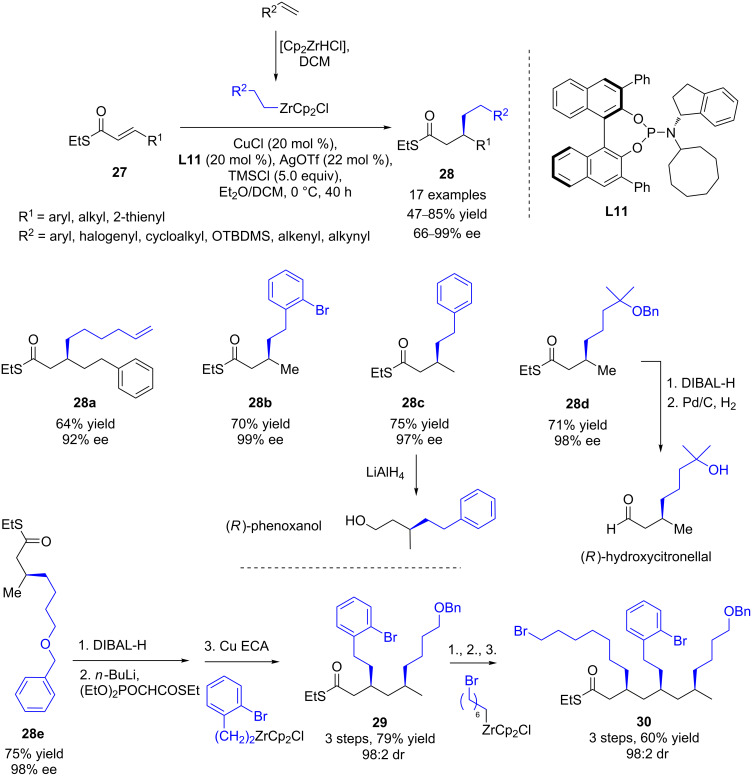
Cu ECA of alkylzirconium reagents to α,β-unsaturated thioesters.

Very recently, Fletcher and Gao reported the first copper-catalyzed ECA of alkylzirconium reagents to α,β-unsaturated thioesters [33]. Starting from diversely functionalized alkenes, the resulting hydrozirconated adducts were reacted with various β-substituted Michael acceptors in the presence of CuCl and the chiral phosphoramidite **L11** (Scheme 12). Remarkably, the corresponding 1,4-products were isolated in moderate to good yields (around 70%) and up to 99% ee. The high versatility of the protocol was illustrated by the synthesis of commercially relevant fragrances (phenoxanol and hydroxycitronellal). Additionally, an efficient iterative route was also described, allowing to produce the highly functionalized deoxypropionate fragment **30** in good overall yields and excellent stereocontrol for all stereogenic centers (up to 98:2 dr).

#### α,β-Unsaturated acylimidazoles

**Scheme 13:**
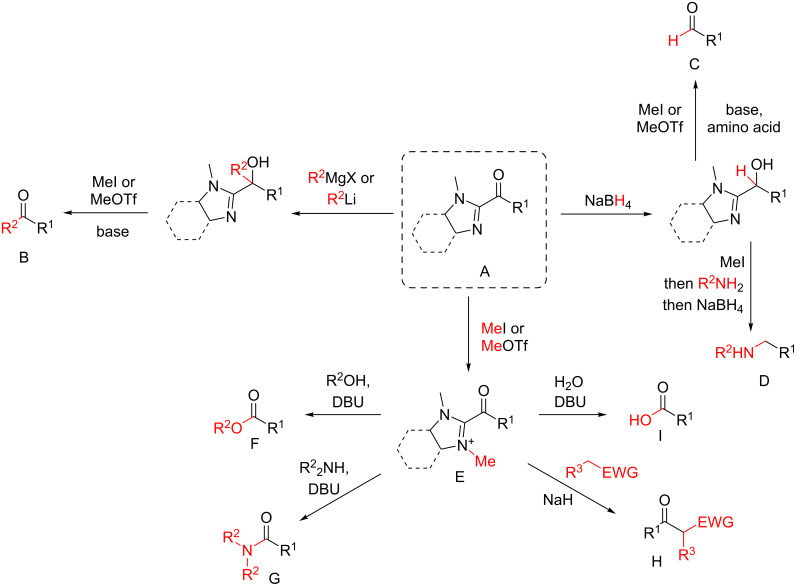
Conversion of acylimidazoles into aldehydes, ketones, acids, esters, amides, and amines.

The pioneering and successful use of α,β-unsaturated acylimidazoles as Michael acceptors in enantioselective catalysis was reported by Evans and co-workers in 2005 [34]. The selected asymmetric transformation was the Friedel–Crafts 1,4-addition involving indole derivatives as nucleophiles, catalyzed by a scandium(III) triflate complex with chiral bis(oxazolinyl)pyridine ligands. As highlighted by Evans, the acylimidazole moiety constituted a privileged surrogate of esters, amides, ketones, and aldehydes. Indeed, this peculiar function, which was readily accessible from the corresponding aldehydes or Weinreb amides, could be efficiently converted into a wide range of carbonyl derivatives, as depicted in Scheme 13.

**Scheme 14:**
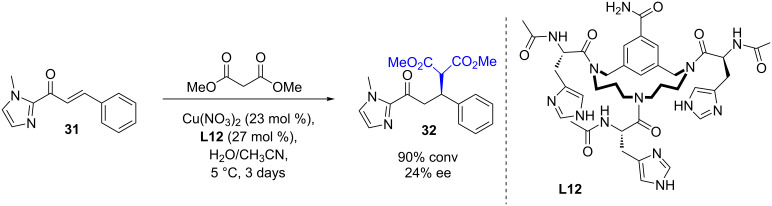
Cu ECA of dimethyl malonate to α,β-unsaturated acylimidazole **31** with triazacyclophane-based ligand **L12**.

The successful use of α,β-unsaturated acylimidazole in Cu ECAs using organometallic reagents has been introduced very recently. Pioneering works in this field were published in 2011 by Roelfes, Liskamp, and co-workers, with the 1,4-addition of dimethyl malonate to cinnamyl 2-acyl-1-methylimidazole (**31**). Unfortunately, in the presence of Cu(NO_3_)_2_ and the triazacyclophane-based ligand **L12**, the product was obtained in a good yield of 90%, but a poor enantioselectivity (24% ee) was observed (Scheme 14) [35].

**Scheme 15:**
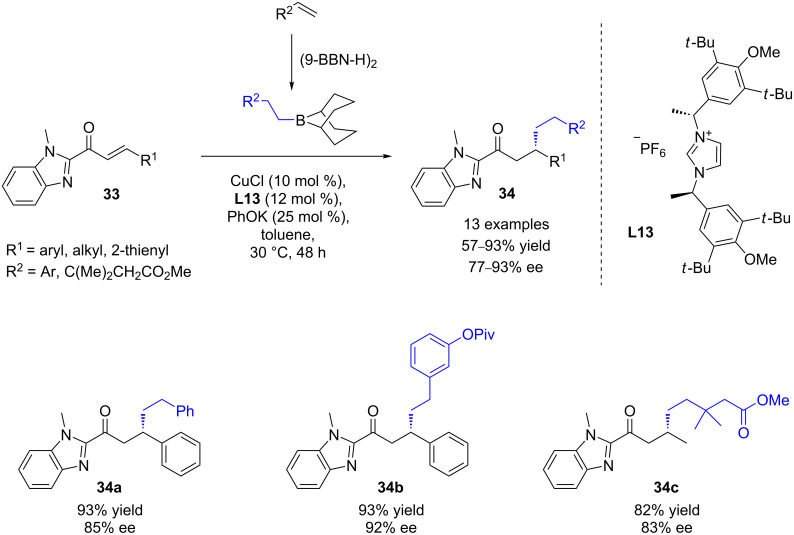
Cu/**L13**-catalyzed ECA of alkylboranes to α,β-unsaturated acylimidazoles.

In 2012, Sawamura and co-workers described the first highly enantioselective copper-catalyzed conjugate addition of alkyl boranes to α,β-unsaturated 2-acyl-1-methylbenzimidazoles **33** [36]. Based on a previous study dealing with the CuCl/IMes-catalyzed addition of various alkylated 9BBN derivatives [37], the authors screened a set of various chiral NHC precursors. The imidazolium compound **L13** appeared to be the most efficient one, affording the desired 1,4 products in high yields (57 to 93%) and excellent ee values (up to 93%, Scheme 15). Advantageously, this methodology was highly tolerant towards a wide range of functional groups, whether on alkylboranes or on substrates.

**Scheme 16:**
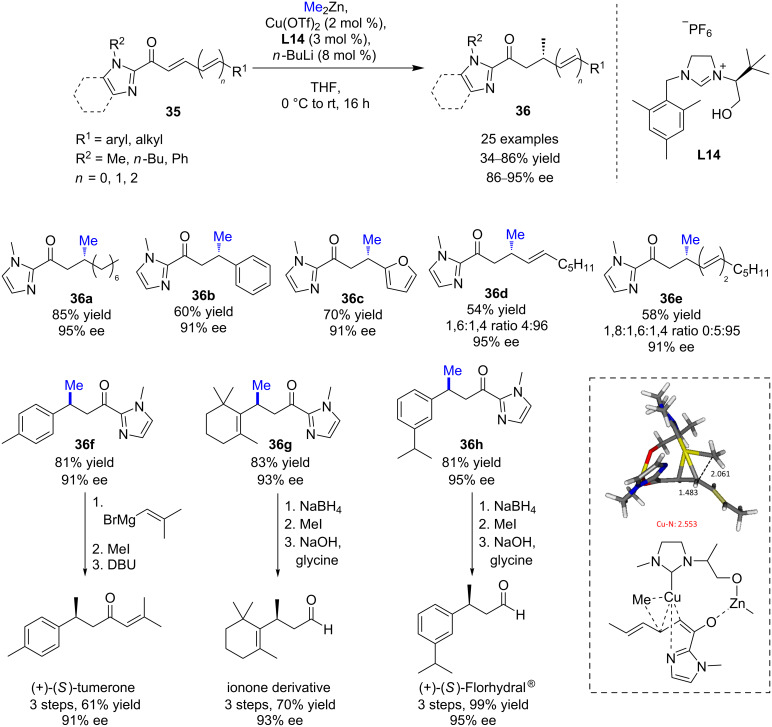
Cu/hydroxyalkyl-NHC-catalyzed ECA of dimethylzinc to α,β-unsaturated acylimidazoles.

In 2015, Mauduit, Campagne, and co-workers set up a highly enantioselective 1,4-addition of dimethylzinc to a wide scope of α,β-unsaturated acylimidazoles **35** [38]. Among the various ligands screened in combination with copper(II) triflate, the hydroxyalkyl-chelating NHC precursor **L14** proved to be the most efficient one, giving the 1,4 product with moderate to good yields (34–86%) and excellent enantioinduction (86 to 95% ee, Scheme 16). The methodology was successfully applied to various extended Michael acceptor systems (dienic or trienic acylimidazoles), leading preferably to the corresponding 1,4 products in moderate to good yields (28–85%) with remarkable regio- (>95%) and enantioselectivities (91–95% ee) [39]. Interestingly, DFT calculations supported the crucial role of the imidazole moiety towards the 1,4-addition (vs 1,6 or 1,8) [39].

Thanks to the efficient post-transformation of the acylimidazole function, the synthetic potential of this methodology was illustrated in the synthesis of relevant molecules, such as a ionone derivative, (+)-*ar*-turmerone, and (+)-Florhydral^®^, which were formed in good yields, without alteration of their optical purity [38–39].

**Scheme 17:**
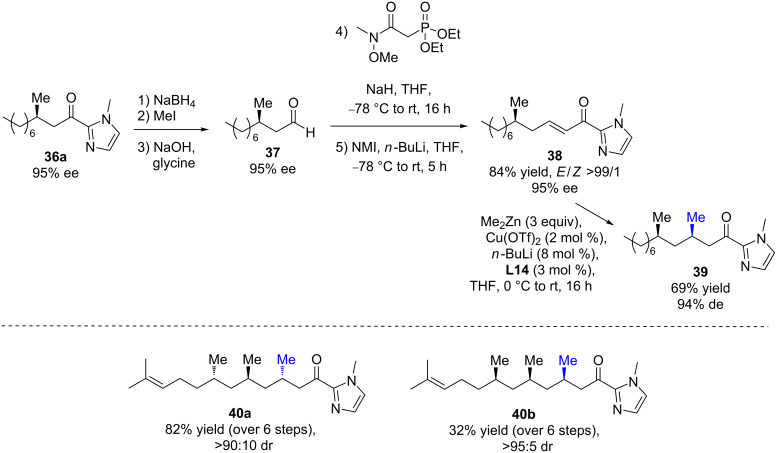
Stereocontrolled synthesis of 3,5,7-all-*syn* and *anti*,*anti*-stereotriads via iterative Cu ECAs.

Moreover, an iterative Cu ECA process allowing the selective introduction of a second methyl stereogenic center was then explored to develop a straightforward access to 1,3-deoxypropionate units, a scaffold ubiquitous in numerous natural products (Scheme 17) [40]. Starting from enantioenriched β-methylated aldehyde **37**, the regeneration of the α,β-unsaturated 2-acyl-1-methylimidazole moiety was performed in high yield and *E*/*Z* selectivity via a two-step protocol. The resulting Michael acceptor was then engaged in an ECA to afford the expected 1,3-dimethyl product in 69% yield and a good diastereomeric excess of 94% (Scheme 17). Following this iterative methodology, the synthesis of 3,5,7-all-*syn*- and *anti*,*anti*-stereotriads **40a**/**b** were successfully achieved in high diastereomeric ratios (up to >95:5) and good overall yields from (*R*)- or (*S*)-citronellal.

More recently, Mauduit, Campagne, and co-workers reported an efficient Cu/Taniaphos-catalyzed β-borylation of an α,β-unsaturated acylimidazole, leading to various enantioenriched β-hydroxy products after oxidation (up to >98% ee) [41]. Interestingly, following the aforementioned iterative ECA strategy, the postfunctionalized chiral acylimidazole **41** derived from (*S*)-citronellal was efficiently converted to highly desirable *anti*,*syn*- and *anti*,*anti-*3,5,7-(Me,OR,Me)-substituted products **42a**/**b**, which were isolated in good yields and excellent diastereomeric ratios (up to >95:5, Scheme 18).

**Scheme 18:**
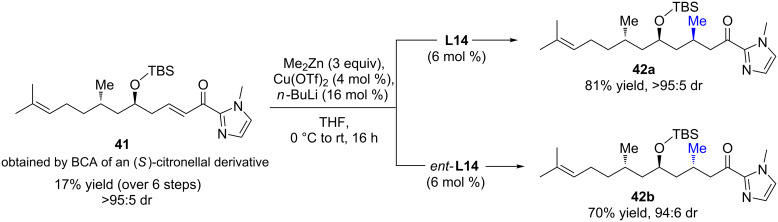
Stereocontrolled synthesis of *anti*,*syn*- and *anti*,*anti*-3,5,7-(Me,OR,Me) units via iterative Cu ECA/BCA.

#### α,β-Unsaturated *N*-acyloxazolidinone, *N*-acylpyrrolidinone, and amide derivatives

Similar to acylimidazole Michael acceptors, the first use of α,β-unsaturated *N*-acyloxazolidinones was also described in asymmetric Friedel–Crafts 1,4-additions catalyzed by chiral copper/bisoxazolidine Lewis acids [42–46]. Thanks to the easy post-transformation of the oxazolidine moiety, the resulting enantioenriched products (up to 99% ee) were successfully converted to relevant molecules, such as *trans*-whisky lactone [43].

**Scheme 19:**
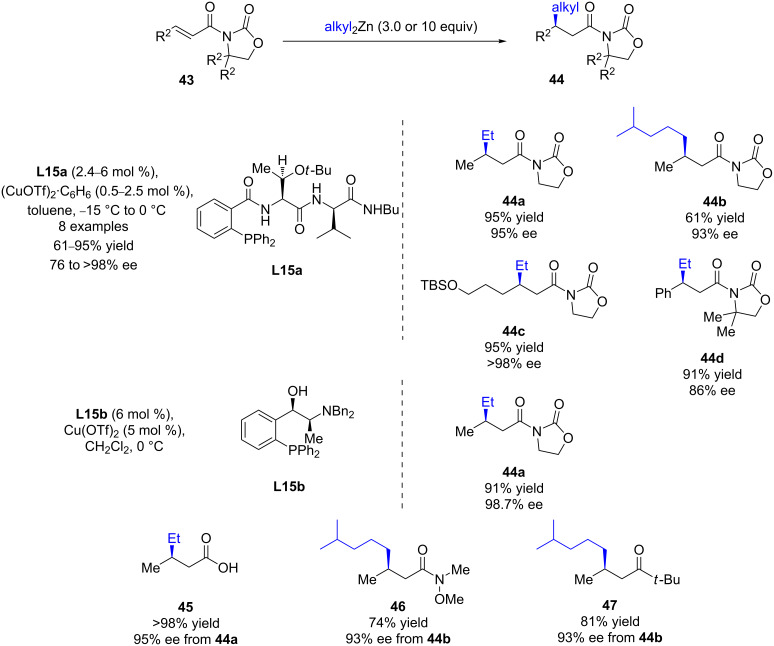
Cu-catalyzed ECA of dialkylzinc reagents to α,β-unsaturated *N*-acyloxazolidinones.

In 2003, Hoveyda and Hird reported the first 1,4-addition of alkylmetal nucleophiles to α,β-unsaturated *N*-acyloxazolidinones (Scheme 19) [47]. The chiral triamidophosphane ligand **L15a** as a copper(I) triflate complex efficiently promoted the catalytic conjugate addition of dialkylzinc species to various *N*-acyloxazolidinone Michael acceptors, in most of the cases with high isolated yields (61 to 95%) and excellent enantioselectivities (up to >98%). Furthermore, the resulting enantioenriched β-alkylated *N-*acyloxazolidinones could be converted to various derivatives (aldehydes, ketones, Weinreb amides, or carboxylic acids) in good yields and without alteration of the ee values. In 2006, aminohydroxyphosphine **L15b** was used as a new designer ligand by Nakamura and co-workers for the addition of diethylzinc to crotonyl *N*-acyloxazolidinone [48]. The 1,4-product was also formed in high enantioselectivity (>98% ee) and high yield (91%).

**Scheme 20:**
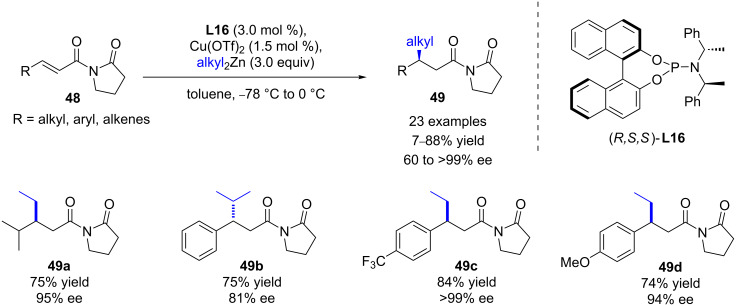
Cu/phosphoramidite **L16**-catalyzed ECA of dialkylzincs to α,β-unsaturated *N*-acyl-2-pyrrolidinones.

The same year, Pineschi et al. evaluated various α,β-unsaturated acyl derivatives for the copper/(*R*,*S*,*S*)-phosphoramidite **L16**-catalyzed addition of diethylzinc (Scheme 20) [49]. Although the Michael acceptor bearing the *N-*acyloxazolidinone moiety that was successfully used by Hoveyda (95% ee) gave a lower enantioselectivity (64%), a more satisfactory enantiocontrol was obtained with the substrate having a 2-pyrrolidinone fragment (87% ee). The scope was then extended to various α,β-unsaturated *N-*acyl-2-pyrrolidinones and dialkylzinc reagents, leading to the corresponding 1,4-products in low to good yields (7–88%), with good to excellent enantioinduction (60 to >99% ee). Trimethylaluminium reagents were also investigated, but unfortunately, only low ee values were observed (20–36% ee), whereas no reactivity was observed with α,β-unsaturated amides.

**Scheme 21:**
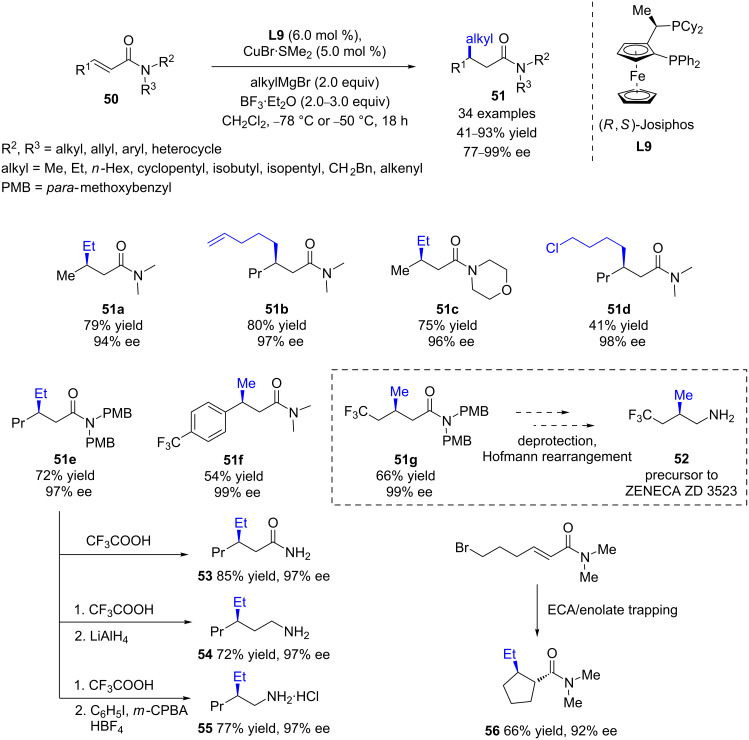
Cu/(*R*,*S*)-Josiphos (**L9**)-catalyzed ECA of Grignard reagents to α,β-unsaturated amides.

Although Pineschi’s conditions were ineffective for amides [49], Harutyunyan and co-workers achieved an important breakthrough by reporting the first enantioselective alkylation of α,β-unsaturated amides [50]. Indeed, due to their poor reactivity compared to other Michael acceptors, catalytic asymmetric conjugate additions of organometallic reagents to *N*,*N-*dialkylenamides remained a real challenge. However, thanks to the synergistic action of the boron-based Lewis acid BF_3_∙Et_2_O, CuBr∙SMe_2_, and the chiral (*R,S*)-Josiphos ligand (**L9**), an efficient (yields up to 86%) and highly regio- and enantioselective (ee values up to 99%) protocol was developed for 1,4-additions of various Grignard reagents to a wide scope of substrates (Scheme 21). Notably, the introduction of methyl and functionalized alkyl groups was performed with remarkable stereoselectivity (97–99%). Furthermore, this catalytic system was easily upscalable (up to 10 g), and the chiral catalyst could be recycled without any loss of efficiency. Unfortunately, although a wide range of Grignard reagents led to excellent results, PhMgBr provided low conversion and the racemic 1,4-product. Additionally, amide substrates featuring a bis(*para*-methoxybenzyl) moiety could be converted into relevant β-alkyl-substituted chiral amines, ubiquitous in numerous pharmaceutical ingredients, such as **52**, a direct precursor of a drug candidate. Moreover, tandem ECA/enolate trapping was also studied, providing the *trans*-cyclopentane product **56** as a single diastereoisomer (92% ee).

**Scheme 22:**
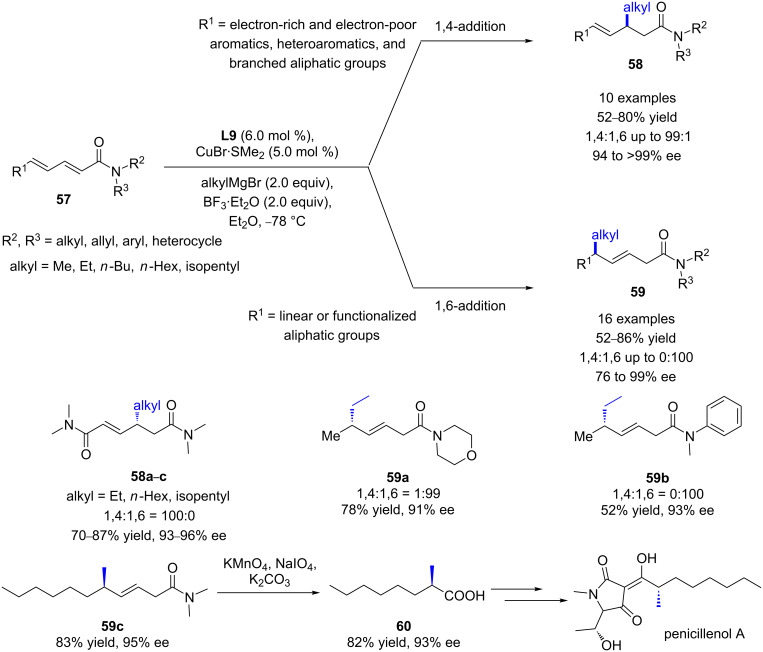
Cu/Josiphos (**L9**)-catalyzed ECA of Grignard reagents to polyunsaturated amides.

In 2018, the same authors reported the 1,6- and 1,4-additions of various Grignard reagents to a wide scope of conjugated dienyl amides (Scheme 22) [51]. Interestingly, the authors observed that the regioselectivity was directed by the substituent in the δ-position of the substrate: dienic amides featuring linear or functionalized aliphatic substituents in the δ-position led predominantly to 1,6-products, whereas those featuring electron-rich and electron-poor aromatic, heteroaromatic, and branched aliphatic substituents in the δ-position afforded preferably the 1,4-products. Importantly, when the morpholine moiety was used as N*-*substituent, the addition of diethylzinc to the enamide afforded the 1,6-addition product with 78% isolated yield and 91% ee. It is worth to underline that the morpholine group could easily allow further postfunctionalizations. Furthermore, thanks to the highly 1,6-enantioselective additions of methylmagnesium bromide (95% ee), this methodology was applied to the synthesis of the natural product penicillenol A by oxidative cleavage of the resulting 1,6 product **59c**, affording the key synthon **60** with a slight erosion of the optical purity (Scheme 22).

#### α,β-Unsaturated *N*-acylpyrroles

**Scheme 23:**
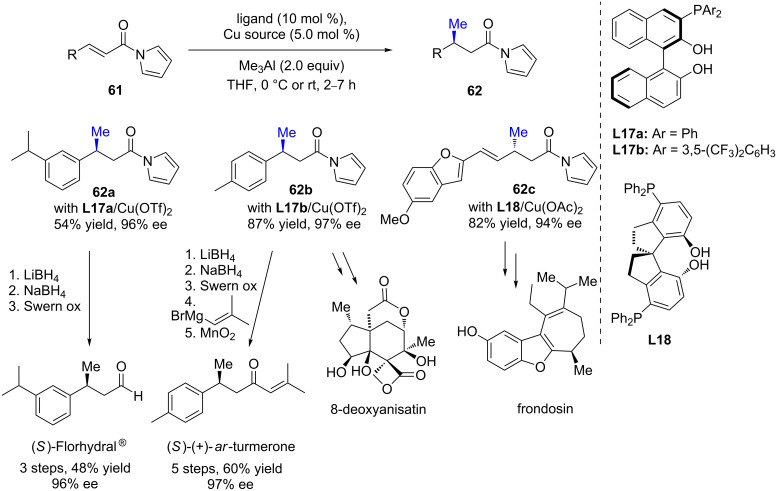
Cu-catalyzed ECA of trimethylaluminium to *N*-acylpyrrole derivatives.

α,β-Unsaturated *N*-acylpyrroles were also investigated as Michael acceptors in enantioselective conjugate additions using organometallic reagents. In 2013, Endo and Shibata described a catalytic system based on multinuclear copper/aluminium complexes and phenolphosphine-based ligands **L17a**/**b** and **L18**, allowing the selective 1,4-addition of trimethylaluminium to three α,β-unsaturated *N*-acylpyrroles with moderate to good yields (54 to 87%) and excellent enantioselectivities (94 to 97% ee, Scheme 23) [52]. This methodology was successfully applied to the synthesis of various natural molecules, such as (*S*)-Florhydral^®^ and (*S*)-(+)-*ar*-turmerone or key intermediates in the synthesis of 8-deoxyanisatin and frondosin.

## Conclusion

The enantioselective Cu-catalyzed conjugate addition of organometallic reagents to Michael acceptors has been extensively studied for many decades and led to remarkable results. However, for a long time, some classes of Michael acceptors (α,β-unsaturated aldehydes, thioesters, acylimidazoles, *N*-acyloxazolidinone, *N*-acylpyrrolidinone, amides, *N*-acylpyrroles) have been neglected to varying degrees, probably owing to their particular reactivity, which led to less impressive results. Nevertheless, these substrates present a high potential in total synthesis, since the chiral products can be easily transformed into various natural compounds. For example, the aldehyde function, which is directly obtained from α,β-unsaturated aldehydes or is accessible through postderivatization of either acylimidazole or thioester functions, is present in many natural compounds and is also a key functional group for many synthetic strategies. Furthermore, some of the functional groups listed above can be converted into various other synthetically useful groups, such as ketones, esters, carboxylic acids, and (Weinreb) amide groups.

More recently, some works were reported, which showed that through a judicious selection of chiral ligands and a fine-tuning of the reactivities of both partners, interesting selectivities could be reached with these more challenging electron-deficient alkenes, including, in some cases, di- or trienic acceptors, which accordingly extends the scope and the synthetic applicability of the method. The potential of the methodology has been illustrated through the efficient conversion of some 1,4-products into various chiral natural products. In addition, iterative procedures leading to chiral 1,3,5-(Me,Me,Me) and 1,3,5-(Me,OH,Me) motifs in a stereocontrolled way were successfully applied from α,β-unsaturated acylimidazoles and α,β-unsaturated thioesters, and thus opening a new field for the total synthesis of natural products. We hope that the results collected in this review will encourage chemists on one hand to continue the search for improved procedures combining simple, easily accessible chiral ligands, lower catalytic loadings, high ee values and productivity, and a wide scope, and on the other hand, to include this highly promising methodology in many synthetic strategies.
